# Medical Devices in the Region of Tuscany, Italy: Definitions of Innovative Devices and Potentially Innovative Devices and Their Impact on Procurement Decisions

**DOI:** 10.7759/cureus.75839

**Published:** 2024-12-16

**Authors:** Andrea Messori, Sabrina Trippoli

**Affiliations:** 1 Health Technology Assessment (HTA) Unit, Regione Toscana, Florence, ITA

**Keywords:** cost-effectiveness analysis, high-cost medical devices, high-technology medical devices, innovativeness, medical devices, potential innovativeness

## Abstract

In the region of Tuscany in Italy, since 2019, medical devices belonging to risk classes 2b, 3, or active implantable have been managed by a multidisciplinary health-technology assessment body initially composed of eight members and extended to 23 members in 2022, thus creating the Centro Operativo. In 2021, an original algorithm for the identification of innovative devices based on objective criteria was developed and formally recognized over the following years. However, since these criteria identified only a small number of innovative devices, we tried to develop another algorithm aimed at identifying a new classification (called "potentially innovative device"), which was intended to be intermediate between full innovation and no innovation. Since preliminary analyses showed that objective criteria were not able to identify this intermediate classification, we relied on the expert opinion of the Centro Operativo for this purpose. In this article, we analyzed all the devices requested by Centro Operativo in the first half of 2024 for a total of 17 devices. Only one met the criteria of innovation and, therefore, was purchased; the other 11 were evaluated as adequate to be purchased based on clinical and/or economic reasons, whereas the remaining six did not. To create an operational relationship between these decisions and the definitions of innovativeness, the Centro Operativo explored a simple model wherein the 11 devices that failed to meet full innovativeness but were judged adequate to be purchased were classified as potentially innovative. In comparison, the remaining six devices were considered not innovative. On the one hand, we report the results of this preliminary experience; on the other hand, we plan to implement this form of management of high-technology devices into a regional regulation that will be applied for the next months in all hospitals of our region.

## Introduction

In the Tuscany region of Italy (3.7 million inhabitants), as of 2019, all medical devices (MDs) classified as risk class 2b, 3, or active implantable have been managed by an ad hoc multidisciplinary health-technology assessment (HTA) body. This committee was initially composed of eight members (Gruppo Regionale sui Dispositivi Medici, GRDM) and was extended in 2022 to a multidisciplinary regional committee of 23 members, thus creating the Centro Operativo. Each HTA assessment focuses on a single device and is based on preparing a mini-HTA report published on the regional website. This mini-HTA report determines whether the MD can be purchased and made available to all public hospitals in our region. Centro Operativo has managed this HTA process for more than two years. All mini-HTA reports are publicly available (at the URL: https://www.regione.toscana.it/-/prodotti-hta). Currently, more than 150 mini-HTA reports have been published on the website, each focused on a new device requested after 2019 by a clinical unit of a public hospital in the region [1]. A positive decision from the Centro Operativo, confirmed by a second committee called the HTA Committee, is mandatory to purchase the requested device. This acquisition has to deal exclusively with devices that had never been purchased before [2-4].

The Centro Operativo works through monthly meetings to produce formal HTA documents. This committee is responsible for editing the mini-HTA report for the requested device and then making a positive or negative decision on the acquisition of the device based on the mini-HTA report. The final step is the formal approval of this decision by the regional HTA committee. Transparency is an important aspect of this device management, as all mini-HTA reports are published on our region's official website, making them publicly available to everyone. In addition to the mini-HTA reports, the website publishes, in full transparency, each acquisition decision made for each of the devices reviewed; these decisions are accompanied by a detailed description of the reasons that led to the decision. Regarding the management of devices in Italy, HTA assessment is performed only at the regional level, as there is no regulatory HTA agency at the national level or the European level. This framework is expected to improve at both national and European levels, but not before January 2027.

An important feature of managing MDs in our region is that, in 2022, an official definition of innovation for high-technology devices was adopted through a regional decree [2,3], which was then put into practice [5]. More specifically, while it was assumed that the procurement of all innovative devices is mandatory, only a minority of devices were initially found to meet the criteria of innovativeness. As a result of our recent experience with the approval of numerous devices that did not meet the criteria for being innovative [1], we focused our retrospective analysis on these approvals of noninnovative devices to identify a subset of objective characteristics that proved to be common to these devices. The aim of this retrospective analysis was to overcome the two-level criterion (innovative vs. noninnovative) that was leading to the approval of too few (innovative) devices and the rejection of too many (noninnovative) devices. For this purpose, using pharmaceuticals as a model [6,7], we started the present study to define a three-level decision algorithm that included the approval of innovative devices, the approval of some noninnovative devices (classified as “potentially innovative” according to the term used for pharmaceuticals), and the rejection of the remaining noninnovative devices.

For this purpose, the starting point of our study was the retrospective review of all the decisions taken by the above HTA body in the first half of 2024, in which a total of 17 devices were assessed [1]. A strength of our project was the availability of the above-mentioned committee of 23 experts, i.e., the Centro Operativo [5], as well as the challenging work this committee has successfully done in the past years. Therefore, our reference point was represented by the “real world” decisions (either positive or negative) taken by the Centro Operativo on the purchase of each of these 17 devices. Based on these real-world decisions, we then explored the characteristics of the devices that received a positive decision compared to those of the devices that received a negative one. The objective was to study retrospectively these 17 devices to evaluate the feasibility of implementing a three-level decision algorithm in which potentially innovative devices are not necessarily defined based on objective criteria.

## Materials and methods

Study design

In the context of the activities carried out by the Centro Operativo, the aim of the present study was to test the hypothesis of establishing the definition of the potentially innovative device as a new intermediate option between innovative and noninnovative. These new definitions imply a three-level decision algorithm (innovative vs. potentially innovative vs. not innovative) as a replacement for the current two-level algorithm. The devices included in this analysis were those evaluated in the first half of 2024, belonging to risk classes 2b, 3, and active implantable. Overall, these three risk classes [8,9] tend to identify all high-technology devices; since 2017, the regulation on risk classes of MDs applies to all countries of the European Union. During this process, the definition of innovation remained unchanged compared to that reported in our previous scientific article [2] and in the regional decree that formally adopted it [3]. To achieve the above objectives, the approved or not approved status of these devices was registered from the mini-HTA reports, together with the specific reasons that led to the specific decision.

The algorithm of innovativeness recognition for high-tech devices in Tuscany

This algorithm was established in June 2022 [3] and is based on the evaluation of the following four points: a) the availability of evidence on the effectiveness and safety of the new device compared to the standard of care for the specific clinical indication; b) the need in clinical practice for the new MD in relation to the regional health care context; c) the reported benefits and risks of the new device; and d) the predicted organizational and economic impact if the new device receives a positive decision.

Regarding the criterion of the availability of a controlled trial comparing the new device with the old device (assumed to be the standard of care for the clinical indication), our algorithm explicitly suggested that this criterion be applied with flexibility. While it is agreed that a randomized trial is the gold standard for a clinical comparison, our algorithm also accepts historical controls to make these comparisons, as well as an indirect comparison, provided that an appropriate report of indirect comparison is available. Finally, our algorithm of innovative device recognition requires that the incremental benefit of the new device be represented by either an improvement of at least 0.15 quality-adjusted life years (QALYs) per patient and/or savings in health care costs of at least €2,000 per day.

From an operational point of view, innovative devices are subject to rapid procurement without competitive tenders, while all other devices are subject to normal procurement (which, in most cases, involves competitive tenders). In fact, the original aim of this definition of innovation adopted in 2022 was to minimize two risks: a) delaying access to innovative devices by subjecting them to a normal procurement path and consequently a slow turnaround time, and b) allowing immediate but useless access to devices that lack any significant advantage.

In the field of MDs, innovation has been much less discussed than in the field of pharmaceuticals. In Italy, the regulation of innovative pharmaceutical products was established in 2017 through a resolution [6] issued by the National Agency for Medicines following the adoption of a national law. According to this regulation, innovative medicines, which are included in a national list [7], are immediately made available to patients, even without formal inclusion in regional formularies. With regard to MDs, as described above, a first attempt to adopt a regulation on innovative devices was made in Italy by the Tuscany region between 2021 and 2022. The Tuscany region was the only one that explored a formal application of this definition through the activities performed by the GRDM up to September 2022 and by the Centro Operativo [5] after September 2022. As previously pointed out, only devices belonging to risk classes 2b, 3, and active implantable were considered. In operational terms, the mini-HTA reports have been prepared exclusively by the Centro Operativo.

The acceptable cost-effectiveness of all approved devices

The regional decree of 2022 that regulated the attribution of innovation [3] contains an important clause that needs to be mentioned: if the real price of a device is particularly high and clearly exceeds a threshold of €60,000 per QALY gained [10,11], the negative criterion for device acquisition prevails even in the case of a fully innovative device. Of course, it was assumed that this negative criterion would also apply to potentially innovative devices.

## Results

Table 1 summarizes the criteria on which our three-level classification of innovativeness is based.

**Table 1:** Classification of devices of risk class 2b, 3, or active implantable according to the definitions of innovativeness adopted by the Centro Operativo in the region of Tuscany in Italy ^*^For this purpose, the availability of a randomized controlled trial is not mandatory and also indirect comparisons are acceptable, which can be based on either the comparison of the new device with historical controls or on other forms of comparative evidence differing from a randomized trial (e.g., measurement of the clinical endpoint in the same subject before and after the implantation) ^**^When both criteria 1 and 2 are met but criterion 3 is not met, the device cannot be classified as innovative ^***^When the benefit is expressed as the cost for avoiding a negative clinical outcome (expressed as a binary endpoint), the cost-effectiveness is considered acceptable if the cost for avoiding the event using the new device is lower than the cost to treat the same event, if not avoided QALY: quality-adjusted life year

Classification	Criteria adopted by the Tuscany region
Innovative	Availability of clinical studies comparing the new device with the standard of care^*^
	Presence of a clinical and/or economic benefit that meets the prespecified threshold (i.e., Case A: incremental clinical benefit ≥0.15 QALYs/patient; and/or Case B: economic or organizational benefit of at least €2,000 deriving from all interventions performed over a day)^**^
	If computable, the cost-effectiveness ratio of the device is below the threshold of €60,000 per QALY gained or per life year gained^***^
Potentially innovative	Expert opinion of a committee of 23 healthcare professionals (the Centro Operativo) who decides to purchase the device
Not innovative	All the devices that fail to meet both criteria reported above

The information recorded from the 17 included devices is presented in Tables 2, 3. Table 2 shows the 11 devices that were approved, while Table 3 shows the six devices that were not approved. The Appendix shows the Internet links to access each of the 17 mini-HTA reports (which are bilingual, Italian, and English). Quite interestingly, the information appearing in Tables 2, 3 clearly shows the heterogeneity of the clinical conditions addressed by these 17 devices.

**Table 2:** Characteristics of the 11 devices that were approved ^*^References in this column refer to the main clinical study on the device; if no reference is reported, information on this point can be found in the mini-HTA report indicated in Table 3 BPH: benign prostatic hyperplasia; HTA: health-technology assessment; IC: indirect comparison made ex-post; nrCT: nonrandomized controlled prospective trial; NT: no trial; RCT: randomized controlled trial; SA: single arm; TURP: transurethral prostate resection; CE: European Community

Device (with identification code)^*^	Target population	Comparator	Type of comparison	Clinical endpoint	Summary of the criteria on which the decision was based
FlowTriever (No. 276) [12]	High-risk and intermediate-risk patients with pulmonary embolism with contraindications to systemic thrombolysis	Systemic thrombolysis or anticoagulant therapy	nrCT	Composite endpoint of multiple causes of unsuccess (e.g., hypotension)	Adequate demonstration of increased effectiveness through a non-randomized controlled prospective trial
Hot Spaxus (No. 303) [13]	Patients suffering from complications of acute or chronic pancreatitis, stenosis of the biliary tract with obstructions that cannot be overcome by retrograde drainage, cholecystitis that cannot be treated surgically	Hot Axios	IC	Severe bleeding (i.e., bleeding requiring transfusion and/or intervention)	Reduced costs and better safety profile estimated through an indirect comparison with the competitor
Circular suturing machine for open surgery (No. 307) [14]	Patients with rectal colic anastomoses are considered technically complex	Conventional circular staplers	IC	Anastomotic bleeding	Despite the increased cost of using the device, this increase is offset by the savings related to the reduced rate of anastomotic leaks and the reduces cost of treatment for this complication
Farawave (No. 309) [15,16]	Atrial fibrillation	Thermal ablation	RCT	No improvement in clinical outcomes	In consideration of the availability of three pulsed-field ablation devices with the same acquisition cost, the decision is meant to be a compromise in which all three devices are made available to the cardiologists; while Farawave is supported by a higher number of clinical studies, the adoption of this advantage is left to the confirmation by individual cardiologists who are allowed to use Farawave to a greater extent than the other two devices
Urolift (No. 289) [17]	Young patients with BPH and retrograde ejaculation in need of fertility preservation	TURP	RCT	BPH6 endpoint recovery (a composite of six elements)	The approval is essentially related to the potential benefit due to the preservation of ejaculation in young patients together with the minimally invasive nature of this device compared to TURP
Signia small	Patients with lung cancer undergoing thoracic surgery	Sutures with larger diameter loaders	NT	-	Although the price of this device is higher than that of the comparator, the approval is based on the potential clinical benefits of the device and the small annual amount needed in our hospitals
Tricvalve (No. 296) [18]	Patients with severe symptomatic tricuspid valve regurgitation at high surgical risk or inoperable	Surgical valve replacement	SA	Reduction in the degree of tricuspid regurgitation	The TricValve system is suitable for a target population at high surgical risk or inoperable with tricuspid valve insufficiency and caval reflux; compared to other devices with a tricuspid indication, the approval derives from a variety of considerations: a) the procedures performed with caval valve implantation have some inherent advantages due to the relative simplicity of the caval anatomy compared to the tricuspid valve and right heart; b) the anatomical and periprocedural imaging requirements for caval valve implantation are less stringent for this device; furthermore, the TricValve device is unique in having CE marking with this indication
Venaseal (No. 297) [19]	Patients with varicose veins in the lower limbs	Radiofrequency intervention	RCT	Freedom from recanalization at 60 months	In patients with varicose veins, the use of cyanoacrylate with Venaseal is comparable to radiofrequency in terms of target vein closure, pain, and recanalization. Compared to surgery, Venaseal demonstrated equal efficacy with regard to closing the target vein, but greater efficacy with regard to reducing pain and the degree of ecchymosis
Cardioband Tricuspid (No. 287-299) used transcatheter annuloplasty [20]	Patients with severe tricuspid valve insufficiency caused by annular dilatation who remain symptomatic on medical therapy and at high risk of surgery	Transcatheter edge-to-edge repair with TriClip®, PASCAL, or off-label MitraClip	IC	Reduction in the degree of tricuspid regurgitation	The Cardioband system is indicated for patients who would otherwise not undergo tricuspid valve repair due to the high surgical risk. In contrast to transcatheter competitors for edge-to-edge valve repair (Pascal and TriClip) that are used when the valve insufficiency is due to valve leaflets, Cardioband is indicated when tricuspid regurgitation is caused by annular dilatation
Optilume (No. 310)	Patients with recurrent urethral stricture resistant to urethrotomy surgical treatment who are candidates for urethroplasty	Uteroplasty surgery	RCT	Success of the intervention	The device has been approved for patients with bulbar urethral stenosis of ≤3 cm in length who are not candidates for urethroplasty, as indicated by the guidelines of the European Urology Association
Neovasc Reducer (No. 315) [21]	Patients with refractory angina undergoing maximal medical therapy and nonrevascularization	Sham procedure	RCT	Quality of life	Cost-effectiveness analyses suggest a favorable profile (especially when data are extrapolated beyond one year) or a borderline profile (if data are limited to the first 12 months). In the light of these considerations, a favorable opinion is expressed, bearing in mind the small annual requirement indicated in the request of the hospital

**Table 3:** Characteristics of the six devices that were not approved ^*^References in this column refer to the main clinical study on the device; if no reference is reported, information on this point can be found in the mini-HTA report indicated in this table ^**^CardioMEMS HF, which was found to meet the criteria for full innovativeness, was not approved because of the application of the clause of an excessive price compared with our regional willingness to pay a threshold of €60,000 per QALY gained ACC: American College of Cardiology; AHA: American Heart Association; ESC: European Society of Cardiology; HTA: health-technology assessment; MRI: magnetic resonance imaging; NT: no trial; NYHA: New York Heart Association; QALYs: quality-adjusted life years; RCT: randomized controlled trial; SA: single arm

Device (with identification code)^*^	Target population	Comparator	Type of comparison	Clinical endpoint	Summary of the criteria on which the decision was based
Endovascular system EkoSonic (No. 126) [22]	Patients with acute high-risk pulmonary embolism who are hemodynamically unstable and patients with intermediate-high-risk pulmonary embolism who present evolution toward hemodynamic deterioration despite appropriate anticoagulant therapy and with three absolute or relative contraindications to the use of systemic thrombolysis (which is the therapeutic strategy of first choice) due to a high hemorrhagic risk	Catheter-directed thrombolysis	RCT	Reduction of systolic pressure in the pulmonary artery	Recent publication of negative clinical studies demonstrating no incremental effectiveness compared with the competitor, along with an increase in costs
Intellis Surescan MRI (No. 301) [23]	Patients with chronic neuropathic pain (failed back surgery syndrome, postoperative pain syndrome, complex regional pain syndrome 1 and 2, arteriopathies, diabetic polyneuropathy, etc.) who with reasonable certainty will have to undergo MRI examination following implantation due to specific comorbidities or particular anamnestic data	Traditional spinal cord stimulation	SA	Low back pain responder rate	The price difference between Intellis SureScan MRI and WaveWriter (about 23,000 vs. 15,000 euros, respectively) is not justified by an increase in any clinical benefits
Fitostimoline PLUS (No. 302)	Patients with a) chronic decubitus ulcers of venous or mixed vascular origin, b) surgical dehiscence, c) inflammatory ulcers, d) first-degree burns, e) second-degree burns; f) posttraumatic injuries, and g) abrasions	Standard gauzes	NT	Wound repair assessed through multiple endpoints	The unfavorable decision is based on insufficient evidence in favor of the device (resulting mainly from the uncertainties in the duration of the treatment) along with an increased cost of the device compared with other medicated gauzes
Motiva Ergonomix (No. 293)	Female patients in need of breast reconstruction; unilateral mastectomy candidates with particular breast conformation (medium-large volume, medium-high ptosis); female patients aged >18 years in need of breast augmentation	Smooth round silicone gel breast implants	SA	Expected reduction in the inflammatory response	Absence of comparative studies vs. other prostheses in terms of efficacy and/or safety. The only partial exception is the study by Sforza et al. [24] (see mini-HTA report) in which Ergonomix prosthesis was compared with the VelvetSurface prosthesis. No differences were observed in the incidence of complications in the subgroup of patients who received implants with a volume between 300 and 499 mL, whereas the device showed a reduced rate of complications in other patients’ subgroups. At the current price levels for this device, the low quality of the clinical evidence and the high price are the main determinants of the negative decision
CardioMEMS HF (No. 305) [25]	Heart failure patients in NYHA Class III admitted at least once in the previous 12 months for heart failure as per ESC and AHA/ACC guidelines	No remote monitoring of the patient	RCT	QALYs	A pilot experience based on 20 patients will be carried out with registration of outcomes at six months. With this exception, the device is not approved^**^
Fixnip (No. 292)	Patients undergoing skin-sparing mastectomy in the absence of an areola-nipple complex and in any case patients undergoing mastectomy with loss of projection of the areola-nipple complex	No other such device is available	NT	Reconstruction of the areola-nipple complex	Since no clinical study describing a documented experience with this device is available, the Centro Operativo makes an unfavorable decision on its purchase

An analysis of Tables 2, 3 shows that the approval decisions were based on a wide range of elements, ranging from the availability of a suitable comparator, the design of the clinical trial, the overall quality of the clinical evidence, the magnitude of the incremental benefit of the new device, etc., and, last but not least, the price of the new device compared to the price of the comparator. Only one (5.7%) of these 17 devices, i.e., CardioMEMS HF, Abbott, Lake County, IL (Table 2), met the criteria for innovation as per the regional decision; however, this device was not approved due to the application of the excessive price clause with respect to our regional willingness-to-pay threshold of €60,000 per QALY gained.

Furthermore, Tables 2, 3 confirm that identifying objective criteria for the definition of potentially innovative and noninnovative devices is very challenging. Although we made numerous attempts to pursue this task (detailed data not shown), none of them was successful. This is easily explained because, as shown in Tables 2, 3, there is a large overlap between the two subgroups in terms of objective selection criteria. For these reasons, in the present paper, we adopted the subjective criterion of the panel of experts method [26] to distinguish potentially innovative devices from noninnovative devices. The Centro Operativo was assumed to be this panel of experts. Finally, to improve the internal consistency of our algorithm and simplify our proposal, we decided to equate the definition of potentially innovative to that of a device whose purchase is authorized and similarly to equate the definition of nonpotentially innovative to that of a device whose purchase is not authorized.

Regarding the devices classified as innovative, in the typical application of this algorithm, only a minority of the devices are generally found to be innovative [27,28]; consequently, the remaining devices classified as noninnovative represent the majority of the total number. This finding was confirmed by our series of 17 devices, in which only one (5.7%), i.e., CardioMEMS HF, met the criteria of full innovation. As a result, this three-level definition of innovativeness is likely to determine an improved management of these MDs.

## Discussion

To better understand the issue at hand (i.e., the definition of a potentially innovative MD), it is important to clarify the various aspects behind the issue itself and to discuss the very definition of innovation in the field of MDs and its degree of acceptance in the Italian context and in the international context. In the Italian context, no formal definition of innovative MD has ever been proposed, with the exception of the present experience made in the region of Tuscany. At the international level, some experiences have been reported, especially from a regulatory point of view. The Breakthrough Medical Devices program of the United States Food and Drug Administration [29] is one such example (with criteria somewhat similar to those used in Tuscany), but there are also similar programs in Australia [30] and Japan [31]. In Europe, France has a similar program called Coverage with Evidence Development [32]. In general, the link between approval and reimbursement is always a critical issue in this area; the Tuscany program brings a kind of automatic coverage, which was initially proposed in the United States for breakthrough drugs and MDs but was quickly suppressed. On the other hand, a common feature of the above experiences is that the innovative device classification is mostly applied to a small proportion of products, as also our experience has shown [27,28].

It should be emphasized that the currently available literature has not been able to identify reliable objective criteria of potential innovation for pharmacological agents, even though these products are supported by much more clinical evidence than MDs. Therefore, the failure of our attempts to objectively classify all potentially innovative devices is not surprising. In Tables 2, 3, the large overlap of information reported in the last column is a clear confirmation of this conclusion. Based on our results, however, it appears that the criteria leading to nonapproval (Table 3), e.g., insufficient number of clinical trials, lack of adequate clinical comparisons, price too high in relation to the magnitude of the clinical benefit, etc., were somewhat more homogeneous than those leading to approval (Table 2); nevertheless, we were unable to formulate an efficient objective proposal for distinguishing potentially innovative from noninnovative devices.

In the decision-making process, relying on expert opinion is generally a well-tested procedure [26]. In the case of the Centro Operativo, two important advantages are the specific HTA expertise of all its members and the large number of members. On the other hand, while the above-mentioned large number of experts participating in this experience is a strength of our study, the rather small number of devices included in this preliminary experience (N = 17) is certainly a limitation.

Compared to the definition of innovative MDs, the definition of potentially innovative devices may be useful in practice, especially if this definition is automatically linked to reimbursement by the national healthcare system. In our preliminary experience, while the search for objective criteria failed, it should, however, be stressed again that a very limited number of devices were involved. Thereafter, a reassessment after at least one or two years of application will be worthwhile. In the meantime, we will pragmatically continue to place potentially innovative devices (identified by our expert panel method, i.e., by our Centro Operativo) in an intermediate category between innovative devices and devices whose purchase is denied for public hospitals. In this context, when more information is available, it will be worthwhile to reassess whether a set of objective criteria can be established for this purpose. On the other hand, the definition of full innovativeness made by the Tuscany region deserves to be kept unchanged, mainly because it seems similar to the definitions already made elsewhere.

## Conclusions

In conclusion, we believe that the method for identifying potentially innovative devices described herein deserves further real-world testing in our regional setting, and we therefore plan to expand our previous regional regulation to formally include the above expert-based method for identifying potentially innovative devices. Thereafter, we will expand this preliminary experience by including further devices in this evaluation over the next years.

## Appendices

**Table 4:** Description of the 17 devices with their respective internet link for consulting the mini-HTA reports; these reports are bilingual (Italian and English) CAC: cyanoacrylate closure; HTA: health-technology assessment

Device (with identification code)	Device description	Link to the mini-HTA report published on the regional website
Devices included in Table 1		
FlowTriever (No. 276)	Thrombus and embolus extraction/aspiration system	https://www301.regione.toscana.it/bancadati/atti/Contenuto.xml?id=5424579&nomeFile=Decreto_n.13540_del_18-06-2024-Allegato-4
Hot Spaxus (No. 303)	Stents with electrocautery delivery system	https://www301.regione.toscana.it/bancadati/atti/Contenuto.xml?id=5424581&nomeFile=Decreto_n.13540_del_18-06-2024-Allegato-2
Circular suturer (No. 307)	Circular suturing machine for open surgery	https://www301.regione.toscana.it/bancadati/atti/Contenuto.xml?id=5424583&nomeFile=Decreto_n.13540_del_18-06-2024-Allegato-1
Farawave (No. 309)	Pulsed field ablation catheter	https://www301.regione.toscana.it/bancadati/atti/Contenuto.xml?id=5409796&nomeFile=Decreto_n.4588_del_05-03-2024-Allegato-3
Urolift (No. 289)	Prostatic urethral lifting system	https://www301.regione.toscana.it/bancadati/atti/Contenuto.xml?id=5413261&nomeFile=Decreto_n.6700_del_27-03-2024-Allegato-1
Signia small (No. 294)	Disposable loader with digital sensor for tissue thickness measurement	https://www301.regione.toscana.it/bancadati/atti/Contenuto.xml?id=5407098&nomeFile=Decreto_n.2935_del_13-02-2024-Allegato-2
Tricvalve (No. 296)	Bicaval transcatheter prosthesis	https://www301.regione.toscana.it/bancadati/atti/Contenuto.xml?id=5407102&nomeFile=Decreto_n.2935_del_13-02-2024-Allegato-3
Venaseal (No. 297) for CAC	Locking system	https://www301.regione.toscana.it/bancadati/atti/Contenuto.xml?id=5407099&nomeFile=Decreto_n.2935_del_13-02-2024-Allegato-1
Cardioband Tricuspid (No. 287) used transcatheter annuloplasty	Tricuspid valve reconstruction system	https://www301.regione.toscana.it/bancadati/atti/Contenuto.xml?id=5413259&nomeFile=Decreto_n.6700_del_27-03-2024-Allegato-2
Optilume (No. 310)	Balloon dilatation urethral catheter with paclitaxel coating	https://www301.regione.toscana.it/bancadati/atti/Contenuto.xml?id=5413257&nomeFile=Decreto_n.6700_del_27-03-2024-Allegato-4
Neovasc reducer (No. 315)	Percutaneously implantable intraluminal vascular device designed to create a permanent and controlled reduction of the coronary sinus lumen in order to increase myocardial perfusion in the presence of ischemic myocardiopathy	https://www301.regione.toscana.it/bancadati/atti/Contenuto.xml?id=5416360&nomeFile=Decreto_n.8521_del_19-04-2024-Allegato-3
Devices included in Table 2		
Endovascular system EkoSonic (No. 126)	System for locoregional thrombolysis using ultrasound catheter	https://www301.regione.toscana.it/bancadati/atti/Contenuto.xml?id=5424580&nomeFile=Decreto_n.13540_del_18-06-2024-Allegato-3
Intellis SureScan (No. 301)	Neuromodulation system for pain treatment	https://www301.regione.toscana.it/bancadati/atti/Contenuto.xml?id=5409797&nomeFile=Decreto_n.4588_del_05-03-2024-Allegato-2
Fitostimoline Plus (No. 302)	Hyaluronated gauze impregnated with Regenerase	https://www301.regione.toscana.it/bancadati/atti/Contenuto.xml?id=5407101&nomeFile=Decreto_n.2935_del_13-02-2024-Allegato-4
Motiva Ergonomix (No. 293)	Sterile silicone breast implants WITH Smooth Silk Surface®	https://www301.regione.toscana.it/bancadati/atti/Contenuto.xml?id=5413258&nomeFile=Decreto_n.6700_del_27-03-2024-Allegato-3
CardioMEMS (No. 305)	Wireless heart failure monitoring system	https://www301.regione.toscana.it/bancadati/atti/Contenuto.xml?id=5416362&nomeFile=Decreto_n.8521_del_19-04-2024-Allegato-1
Fixnip (No. 292)	Implantable solid silicone hypodermic implant intended to be placed under the skin in the subcutaneous fat for aesthetic improvement of the female nipple areola	https://www301.regione.toscana.it/bancadati/atti/Contenuto.xml?id=5409798&nomeFile=Decreto_n.4588_del_05-03-2024-Allegato-1
